# GloSEM: High-resolution global estimates of present and future soil displacement in croplands by water erosion

**DOI:** 10.1038/s41597-022-01489-x

**Published:** 2022-07-13

**Authors:** Pasquale Borrelli, Cristiano Ballabio, Jae E. Yang, David A. Robinson, Panos Panagos

**Affiliations:** 1 Department of Science, Roma Tre University, 00146 Rome, Italy; 2 Department of Earth and Environmental Sciences, University of Pavia, 27100 Pavia, Italy; 3 Department of Biological Environment, Kangwon National University, Chuncheon, 24341 Republic of Korea; 4 European Commission, Joint Research Centre (JRC), Ispra, Italy; 5 UK Centre for Ecology and Hydrology, Environment Centre Wales, Bangor, LL57 2UW United Kingdom

**Keywords:** Environmental impact, Climate sciences

## Abstract

Healthy soil is the foundation underpinning global agriculture and food security. Soil erosion is currently the most serious threat to soil health, leading to yield decline, ecosystem degradation and economic impacts. Here, we provide high-resolution (ca. 100 × 100 m) global estimates of soil displacement by water erosion obtained using the Revised-Universal-Soil-Loss-Equation-based Global Soil Erosion Modelling (GloSEM) platform under present (2019) and future (2070) climate scenarios (i.e. Shared Socioeconomic Pathway [SSP]1–Representative Concentration Pathway [RCP]2.6, SSP2–RCP4.5 and SSP5–RCP8.5). GloSEM is the first global modelling platform to take into account regional farming systems, the mitigation effects of conservation agriculture (CA), and climate change projections. We provide a set of data, maps and descriptive statistics to support researchers and decision-makers in exploring the extent and geography of soil erosion, identifying probable hotspots, and exploring (with stakeholders) appropriate actions for mitigating impacts. In this regard, we have also provided an Excel spreadsheet that can provide useful insights into the potential mitigating effects of present and future alternative CA scenarios at the country level.

## Abstract (summary)

**Table:** 

Measurement(s)	Soil displacement by water erosion (t/ha/year)
Technology Type(s)	Grid-based GIS modelling
Sample Characteristic - Environment	Soil system
Sample Characteristic - Location	Global

## Background & Summary

Ploughing activities and unsuitable agricultural practices are the primary causes of accelerated rates of soil displacement (or soil loss^1^) by water erosion, which is recognised as having detrimental effects on agricultural production and reducing ecosystem functioning^2^. Because we live on a cultivated planet, where agriculture covers ~38% of the Earth’s ice-free land^3^, accelerated water erosion represents a major socioeconomic and environmental threat through its several on-site and off-site effects, such as pollution in our waterways, dam siltation, eutrophication phenomena and the contamination of coastal and marine ecosystems^4,5^. In the context of soil being globally poorly governed^6^, with conservation farming still playing a marginal role (estimated to cover 9% to 15% of croplands worldwide, equal to ca. 122–215 Mha^7^), seeking to gain a better understanding of global soil erosion dynamics is important in order to: (i) help decision-makers in developing new interventions and farming practices that are better for the environment and reduce the global footprint of agriculture on ecosystems^8^; and (ii) strengthen knowledge-sharing with earth-system modellers working on the soil erosion implications of sediment, nutrient and carbon cycling^9^.

The US Department of Agriculture (USDA) developed the Revised Universal Soil-Loss Equation (RUSLE)^10^, a deterministic and empirically based prediction approach that developed from a statistical analysis of more than 10,000 plot-years of basic runoff and soil displacement data covering a large variety of North American landscape conditions. Today, RUSLE-based algorithms have been applied in over 100 countries worldwide and are by far the most widely applied soil-erosion prediction models, globally (~41% of the total, according to the global review and statistical analysis of Borrelli *et al*.^11^). The estimates from Global Soil Erosion Modelling (GloSEM) rest on the RUSLE prediction approach implemented in a GIS environment by means of statistical operations and advanced spatial interpolation techniques. We recognise that using an empirically based prediction tool, underpinned by data-driven assumptions, in areas outside the range of the original estimates (e.g. tropical, sub-Arctic and tundra regions) may considerably reduce the local accuracy of the model. However, considering the proven capacity of RUSLE-based models to overcome their empirical origins^12^, the current lack of better-performing models^13,14^, and the need to be able to predict the possible impacts of global change on soil erosion, we argue that this physically plausible empirical method for predicting soil erosion through water erosion represents a legitimate approach to narrowing the current gap in our knowledge and supporting targeted soil-conservation efforts aimed at mitigating soil erosion. Studies detailed in the literature describe the methodological development of the GloSEM framework under present^15^ and future^9^ land-use and climate conditions.

However, GloSEM is not the only modelling approach presented in the literature that quantitatively estimates soil displacement by water erosion on a global scale. Several global modelling approaches have been developed, all of them using RUSLE-based algorithms^16–23^. While these approaches differ in terms of degree of complexity, they all tend to have lumped and coarse-resolution (ca. 10–60-km cell size) modelling schemes that lack the ability to statistically represent the conditions of local farming systems and the mitigation potential that can derive from conservation agriculture (CA) (further details are given in Borrelli *et al*.^15^). This limits the suitability of most water-erosion prediction models to address policy questions, and to support policy-makers in exploring the geographical extent of the problem, deal with hotspots, and work with stakeholders to mitigate on-site and off-site impacts.

**Fig. 1:**
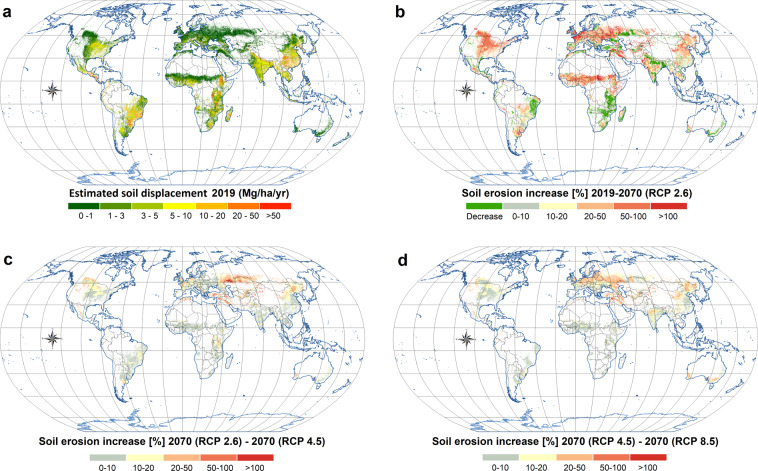
Soil erosion estimates predicted through GloSEM 1.3 for global croplands: (**a**) soil erosion rates divided into seven classes, according to the European Soil Bureau classification; (**b**–**d**) changes in annual average soil erosion between 2019 and 2070 for three distinct RCP greenhouse-gas trajectories. The changes exclusively refer to effects from the climate change projections. For these simulations, the 2019 croplands were used. (**b**–**d**) share the same legend.

**Fig. 2:**
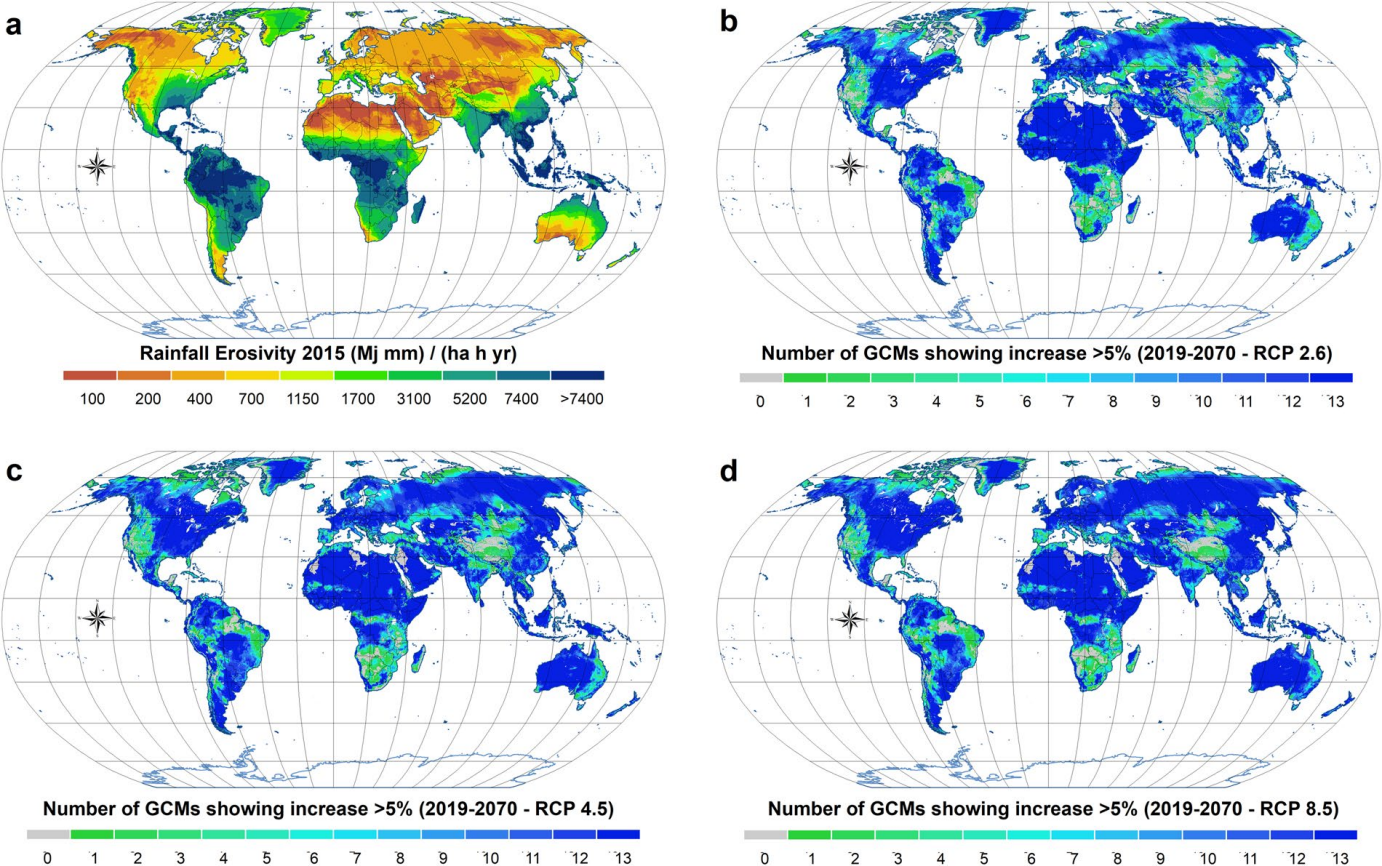
Global rainfall erosivity: (**a**) erosivity classes subdivided according to quantiles; (**b**–**d**) number of GCMs showing changes greater than 5% between 2019 and 2070.

**Fig. 3:**
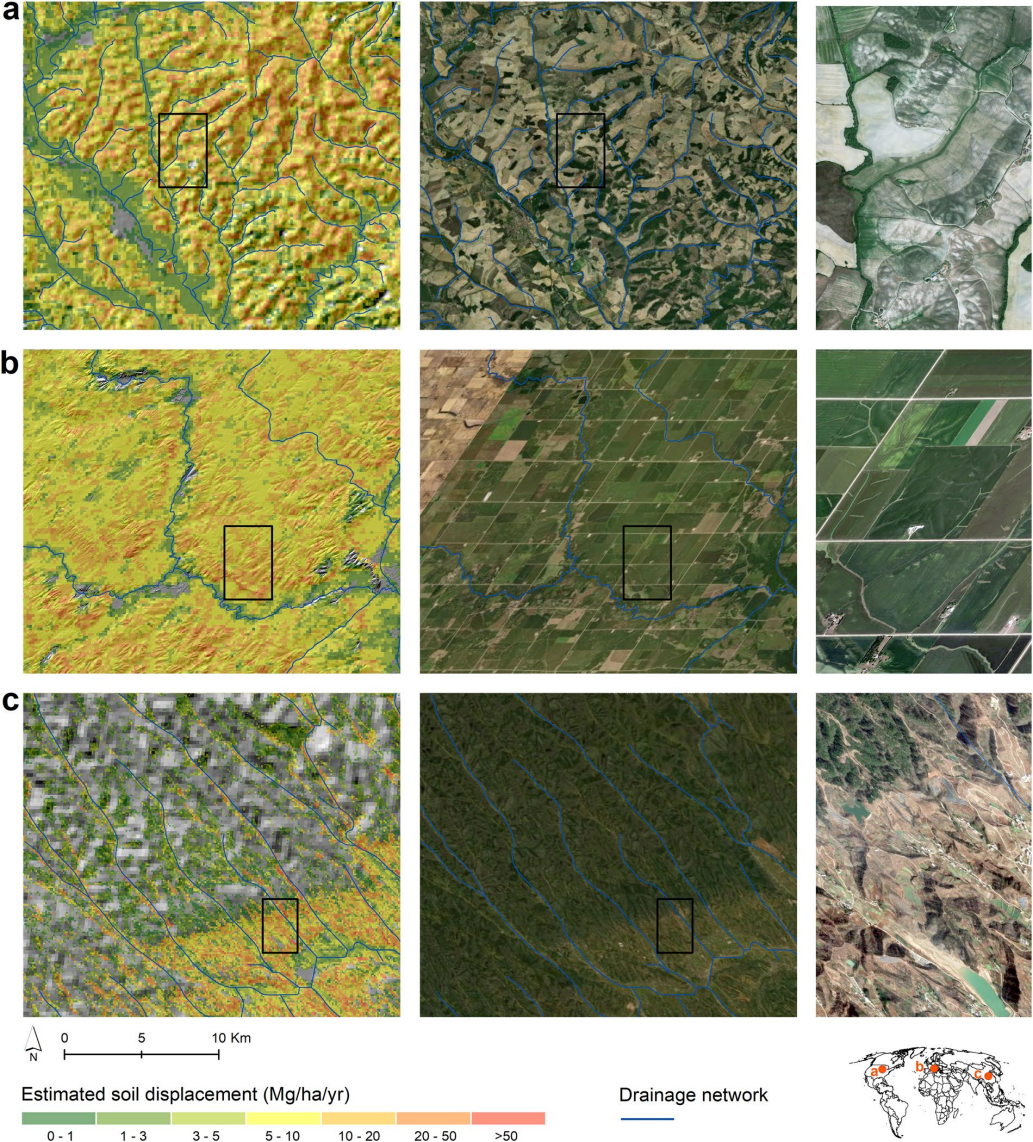
GloSEM estimates in three locations showing signs of susceptible to soil erosion by water in (**a**) Italy (Asciano, Tuscany – 11.49E; 43.25 N), (**b**) USA (Albion, Iowa – 93.09 W; 42.11 N) and (**c**) China (Fangxian, Hubei – 110.71E; 32.13 N). The figures on the left column report the estimates of soil displacement superimposed on a hillshade. The figures on the central column show aerial images (Google Image) for the same locations already reported in the figures on the left column. The figures on the right column are a magnification of the panels reported in black.

The outcomes stemming from GloSEM have been used by researchers in adjacent disciplines to map the world’s free-flowing rivers^24^, quantify the negative cascading effects of soil erosion on nutrient loss^25^, create a link between soil erosion and economic models^26^, measure land degradation^27,28^, and consider alternative strategies for coral-reef conservation^29,30^, among other things. In response to a rising demand for GloSEM data-sharing, we are hereby releasing the latest GloSEM 1.3 estimates for global croplands. We present a gridded geo-dataset (GeoTIFF format) of soil displacement by water erosion, expressed as a mass of soil per unit area and time (Mg ha^−1^ yr^−1^), obtained by running GloSEM coupled with the cropland map from the global land-cover layers released by the European Space Agency (ESA) and the Copernicus Global Land Service land cover (CGLS-LC100–100 spatial resolution) Collection 3 product based on PROBA-V and Sentinel-2 satellite imagery^31^. Present (2019) (Fig. 1a) and future (2070) (Fig. 1b–d) soil displacement under different climate scenarios (Shared Socioeconomic Pathway [SSP]1–Representative Concentration Pathway [RCP]2.6, SSP2–RCP4.5 and SSP5–RCP8.5) are being released on a 100 × 100-m grid-cell basis, covering the croplands of 199 countries (~1.4 billion ha). The descriptive statistics are provided in Table 1 and S1, while Fig. 2 illustrates the present and future rainfall erosivity dynamics. Figure 3 illustrates the detailed modelling results for three areas in Italy, US and China.

**Table 1:** Descriptive statistics of continental and global soil displacement estimates for the 2019 and 2070 scenarios.

	Area	Total displacement						Displacement rate	
		2019	RCP 2.6	RCP 4.5	RCP 8.5	2019	RCP 2.6	RCP 4.5	RCP 8.5
	Million ha	Pg yr^−1^				Mg ha^−1^ yr^−1^			
Africa	248.2	4.3	4.9	5.1	5.2	17.1	19.7	20.5	20.9
Asia	500.9	6.4	7.7	8.0	8.5	12.8	15.3	16.0	17.1
Europe	251.5	0.7	0.9	0.9	1.0	2.6	3.5	3.7	4.1
North America	205.2	2.3	2.9	3.0	3.1	11.0	14.3	14.7	15.2
Oceania	34.2	0.1	0.1	0.1	0.1	2.7	3.1	3.2	3.5
South America	158.0	3.5	3.9	4.0	4.1	22.0	24.5	25.6	25.7
Total	1398.0	17.2	20.4	21.2	22.1	12.3	14.6	15.2	15.8

## Methods

The GloSEM platform follows the same principles of most RUSLE-type models or more-complex catchment-scale process-based models, with a driving force (erosivity of the climate [R-factor], expressed in MJ mm h^−1^ ha^−1^ yr^−1^), a resistance term (erodability of the soil [K-factor], expressed in Mg h MJ^−1^ mm^−1^), and other factors representing the farming choice, such as topographical conformation of the field (dimensionless LS-factor), cropping system (dimensionless C-factor) and soil conservation practices (dimensionless P-factor). The long-term annual average soil displacement (Mg ha^−1^ yr^−1^) estimates rest on a multiplicative equation (Eq. [1]) of these environmental factors:

1 $${\rm{A}}={\rm{R}}\cdot {\rm{L}}\cdot {\rm{S}}\cdot {\rm{K}}\cdot {\rm{C}}\cdot {\rm{P}}$$

Using a GIS-based gridded modelling approach applied to a RUSLE-type algorithm, such as GloSEM, corresponds to the hypothesis that each cell is independent of the others with respect to soil displacement. The provided estimates of soil displacement by water erosion refer to the amount of sediment displaced within each cell. The model does not, in any way, include areas of slope that experience net deposition over the long term^1^. This is because the displaced soil amount is not routed downslope across each cell, from hill slope to sink area or riverine system, through a transport/deposition capacity module. This RUSLE-based soil displacement prediction scheme is preferred to other process-based physical models because the latter require a large number of input data that are not yet mature enough for global-scale applications.

### Model parametrisation

The six environmental factors described in Eq. (1) were computed as follows.

### Land-cover and management factor

The (dimensionless) C-factor measures the combined effect of the interrelated soil-cover and management variables on the soil erosion process. Here, for its spatial computation, we followed the pathway developed in previous GloSEM applications^9,32^. However, in this case, the built-in GloSEM probabilistic land-use-allocation module was substituted with an external cropland layer (reference year 2019)––the CGLS-LC100 Collection 3, developed by the ESA and the CGLS^31^. The CGLS-LC100 Collection 3 comprises a set of annual global land-cover maps (2015–2019), with an overall 80% accuracy, obtained using PROBA-V and Sentinel-2 satellite imagery, and more than 150,000 crowd-sourced training points. The decision to use this new ESA product to represent the global cropland in this new version of GloSEM 1.3 rested on: (i) the high mapping accuracy of the new ESA product; (ii) the high level of agreement with the FAOSTAT cropland information at the global and continental scale; and (iii) its capacity to describe cropland-cover fractions (%) and, in turn, define spatial locations where cropland is mixed with pasture and natural vegetation. The original approach of GloSEM to describe crop rotations was not changed, and it still relies on the 12 years (2001–2012) of crop yields obtained from the FAOSTAT database (https://www.fao.org/faostat/en/#data) for each considered country. The cropland C-factor (*C*_*CROP*_) values were computed at the sub-national level for each of 3,252 administrative regions, as follows:

2 $${{\rm{C}}}_{{\rm{CROP}}}=\mathop{\sum }\limits_{{\rm{n}}={\rm{1}}}^{{\rm{13}}}{{\rm{C}}}_{{\rm{CROPn}}}\cdot \left[ \% \right]{{\rm{Region}}}_{{\rm{CROPn}}}$$

Where, C_CROP_ represents the overall C-factor estimated for each sub-country administrative unit layers (FAO GAUL), C_CROPn_ is the individual C-factor for each of the 170 crops considered (FAOSTAT), and [%] Region_CROPn_ represents the share of each crop in the arable land of the given FAO GAUL administrative unit. The [%] Region_CROPn_ values were assessed through a statistical downscaling of the national crop statistics using the spatially explicit harvested areas proposed by Monfreda *et al*.^33^, combining national-, state- and county-level census statistics with remote-sensing data (for 15,990 administrative units)^34^. The global cropland obtained from the global land-cover map for the reference year 2019 was also applied to the 2070 scenarios. Accordingly, the extent of the global cropland in both temporal scenarios overlapped precisely and consistently, and was equal to ~1.4 billion ha. The regional C_CROP_ values were also kept the same for both temporal scenarios. However, in the Excel spreadsheet we provide further data concerning the country-scale extension of cropland, and estimates computed according to the future land-use/cover scenarios from the integrated assessment model given in the Land-Use Harmonization (LUH2) database^35^—that is, SSP1–RCP2.6 derived from IMAGE, SSP2–RCP4.5 derived from MESSAGE-GLOBIOM and SSP5–RCP8.5 derived from REMIND-MAGPIE. While in the present study future land-use change is not considered, readers can use the Excel spreadsheet (Appendix A) to perform country-scale estimates of soil displacement under alternative scenarios (Table S1). In the future, we plan to release refined GloSEM input data with the purpose of allowing readers to perform their own spatially explicit estimates.

### Rainfall-runoff erosivity

The average annual rainfall-runoff erosivity (R-factor, MJ mm h^−1^ ha^−1^ yr^−1^) is spatially defined using a Gaussian process regression (GPR) geostatistical model for both present^36^ and future^9^ climate conditions. The GPR model established a statistical relationship between the R-factor point values (from 3,625 meteorological stations, associated with the Global Rainfall Erosivity Database [GloREDa], in 63 countries that provide sub-hourly [61%] and hourly [39%] pluviographic data) and a set of WorldClim^37^ climatic data (version 2.1, 30 seconds spatial resolution), acting as spatially exhaustive covariates. The R-factor values for every erosive rainstorm^36^ were calculated adopting the methodology suggested in *USDA Handbook No. 703*^10^, while the USDA’s Rainfall Intensity Summarization Tool was used to facilitate the computation phase of the individual erosive rainstorms. The R-factor values (MJ mm ha^−1^ h^−1^ yr^−1^) from each of the 3,625 meteorological stations resulted from the following equation:

3 $$R=\frac{1}{n}\mathop{\sum }\limits_{j=1}^{n}\,\mathop{\sum }\limits_{k=1}^{mj}\left(E{I}_{30}\right)k$$

Where, EI_30_ is the rainfall-runoff erosivity of a single event, k; n expresses the number of years observed; and mj expresses the erosive events during a given year, j. The WorldClim climatic data, acting as spatially exhaustive covariates in both the present and future climate scenarios, were: (i) average monthly precipitation; (ii) average minimum and maximum monthly precipitation; (iii) average monthly temperature; (iv) precipitation in the wettest month; (v) precipitation in the driest month; and (vi) precipitation seasonality. It is worth mentioning that, for the future (2070) scenarios, the R-factor values were estimated using the climate projections from 14 general circulation models (GCMs) and considering three different SSPs and RCPs (i.e. SSP1–RCP2.6, SSP1–RCP4.5 and SSP5–RCP8.5). This yielded a total of 43 possible future scenarios.

### Soil erodability

The K-factor (Mg h MJ^−1^ mm^−1^) spatially defines the susceptibility of the soil to be eroded. Here, it was algebraically defined (Eq. [4]), based on certain intrinsic soil properties, according to the methodology proposed by Wischmeier *et al*.^38^. Some of the required soil properties (i.e. texture, organic matter and coarse fragmentation) were obtained from the International Soil Reference and Information Centre SoilGrids database at a 1-km spatial resolution^39^. Further soil properties, such as soil structure and permeability, were derived according to the methodology proposed for the GloSEM soil erodability map^32^, which combines textural characteristics (M––percentage of the silt-plus-fine-sand fraction, multiplied by 100, minus the clay fraction), organic matter (OM, in %), soil structure (s) and permeability class (p):

4 $$K=\frac{\left(2.1\times 1{0}^{-4}\;{M}^{1.14}\;\left(12-OM\right)+3.25\left(s-2\right)+2.5\left(p-3\right)\right)}{100}\cdot 0.137$$

### Slope length and steepness factor

The (dimensionless) LS-factor represents the influence of the terrain on the surface runoff and sediment transport capacity. It was computed using the two-dimensional GIS-based approach proposed by Desmet and Govers^40^. The slope and upslope contributing area were calculated using hole-filled Shuttle Radar Topography Mission (SRTM) and Terra Advanced Spaceborne Thermal Emission and Reflection Radiometer (ASTER) Global Digital Elevation Model (GDEM) v2 data with a 3-arc-seconds spatial resolution (ca. 90 m). Due to the large amount of processed data, the computational operations were carried out using the Saginaw Area (SAGA) GIS interface in R statistical computing software. We created 870 tiles of ca. 500 × 500 km.

### Conservation support-practice factor

The (dimensionless) P-factor expresses the ratio of soil displacement using a conservation support practice, such as contouring, strip-cropping, terracing or subsurface drainage. Values for the support-practice P-factor are difficult to report above the field-scale due to the lack of spatial information, and they are not taken into account in the vast majority of regional- and global-scale assessments. In GloSEM^9,32^, the possible mitigation effects of soil conservation rely on the CA data provided by 54 countries to the UN Food and Agriculture Organization’s (FAO’s) AQUASTAT database. These countries regularly communicate the proportion of their cropland managed in accordance with the three FAO CA standards––minimum soil disturbance, organic soil cover and crop rotation/association. Overall, these countries cover 73% of the global cropland. For the remaining 27%, the continental-level CA was considered. For areas under CA, we assumed a reduction in soil erosion ranging between 45% and 70% compared to conventional agriculture. Customised conservation scenarios can be simulated using the provided Excel spreadsheet.

### Heterogeneity of input data

GloSEM versions 1.1 and 1.2 came with a spatial resolution of ca. 250 m at the equator. This resolution was set to be consistent with the satellite data of the Moderate Resolution Imaging Spectroradiometer (MODIS), which constituted a key input for the computation of the Land-cover and management factor (C), the spatial interpolation of the rainfall-runoff erosivity factor (R), and the spatial interpolation of the soil erodability factor (K). Accordingly, all these GloSEM input data were spatially optimized and consistently multiples of the base unit of 250 m defined by the MODIS data. The slope length and steepness factor (LS) was computed at 90 m native resolution (SRTM 3 arc-seconds data) and resampled to 250 m. The resampling caused a loss of detail that we consider within the tolerance threshold.

In the new GloSEM version 1.3, the adoption of the 100 m land-use data provided by ESA caused (i) a slight misalignment of the gridded structure and (ii) the loss of the perfect multiples structure guaranteed from the former 250 m MODIS-based spatial resolution. Despite not being optimal, in our view the increase in heterogeneity of input data due to the change in land-use product is overcompensated for, by the relevant increase in geographical representativeness provided by the ESA fractional (%) cropland layer. A future refinement of the other input data of GloSEM to 100 m spatial resolution is to be considered desirable.

### Error estimate

A summary of error propagation in the present and future predictions was calculated considering: (i) the uncertainty of the spatial predictions estimated using a Markov Chain Monte Carlo approach; (ii) the uncertainty of estimating the area under CA (considered only for the 2015 scenario); (iii) the uncertainty related to the effectiveness of the CA practices using a Monte Carlo method; and (iv) the uncertainty in regional-rainfall-intensity–kinetic-energy relationships. A further uncertainty (v), related to the variation found in the 14 different GCMs, was also considered. The error propagation is expressed as the square root of the sum of squares of the different uncertainties.

## Data Records

The GloSEM 1.3 dataset, containing present and future scenarios, is being made available via the European Soil Data Centre (ESDAC) (https://esdac.jrc.ec.europa.eu/content/glosem) and Figshare^41^ data repositories. The files are being provided in GeoTIFF format, which is a common GIS extension, storing georeferenced data accessible using GIS open-source software. Concerning the coordinate systems, all data are referenced to the World Geodetic System (WGS84). The cell size is 0.00099 decimal degrees, which is equivalent to ~100 m close to the Equator. A total of five layers is being released. Four of them provide estimated rates (Mg ha^−1^ yr^−1^) of soil displacement by water (SOIL_DISPLACEMENT_ESTIMATE_“*scenario*”.tif), while the last layer provides the fractional (%) cropland cover (ESA100_Cropland_fraction.tif). Future updated modelling outcomes will also be made available via the same ESDAC repository. Further details about the layers are provided below.

- SOIL_DISPLACEMENT_ESTIMATE_2019.tif––This scenario considers the ESA’s global cropland (reference year 2019; 1.4 billion ha) and the recent climate scenarios described by Panagos *et al*.^36^ and Borrelli *et al*.^15^.
- SOIL_DISPLACEMENT_ESTIMATE_2070_SSP1–RCP2.6.tif––This scenario considers the ESA’s global cropland (reference year 2019; 1.4 billion ha) and the future (2070) climate scenario obtained from 14 GCMs (WorldClim) for the scenario RCP2.6 (CMIP5) (more details are given in Borrelli *et al*.^9^).
- SOIL_DISPLACEMENT_ESTIMATE_2070_SSP2–RCP4.5.tif––This scenario considers the ESA’s global cropland (reference year 2019; 1.4 billion ha) and the future (2070) climate scenario obtained from 14 GCMs (WorldClim) for the scenario RCP4.5 (CMIP5) (more details are given in Borrelli *et al*.^9^).
- SOIL_DISPLACEMENT_ESTIMATE_2070_SSP5–RCP8.5.tif––This scenario considers the ESA’s global cropland (reference year 2019; 1.4 billion ha) and the future (2070) climate scenario obtained from 14 GCMs (WorldClim) for the scenario RCP8.5 (CMIP5) (more details are given in Borrelli *et al*.^9^).
- ESA100_Cropland_fraction.tif––Global land-cover map (expressed in %) of the ESA and CGLS (CGLS-LC100 Collection 3) for the reference year 2019.

## Technical Validation

As explained by Auerswald *et al*.^42^ and Borrelli *et al*.^15^, the *sensu stricto* validation of regional or larger-scale applications of RUSLE-based models, such as GloSEM, is challenging due to the lack of long-term field-scale measurements. Therefore, GloSEM is presented with a set of procedures to provide insights that support the plausibility of the global estimates, including: (i) uncertainty of the spatial predictions; (ii) a cross-comparison of results with continental studies; (iii) a comparison of results against empirical data; (iv) a comparison of results with river sediment load; (v) a comparison of results with past UN global assessments; (vi) a comparison of results from field/plot measurements; (vii) an uncertainty estimate of the climate erosivity; (viii) a GPR forecasting-capacity analysis; and (ix) a sensitivity analysis to determine the influence of the input parameters (see details in Borrelli *et al*.^9^). Here, we focused our attention on the GloSEM cross-comparison with continental-scale studies and the forecasting capacity of the GPR approach to predict future rainfall erosivity.

### GloSEM cross-comparison with continental-scale studies

A cross-comparison of the GloSEM results was performed, comparing aggregated regional values of predicted soil erosion against the values obtained by regional soil-erosion assessments. The goal of this analysis was to assess the plausibility of the quantitative soil-erosion estimates. The first regional comparison was made with the cropland of the US. According to the National Resources Inventory (NRI) of the USDA^43^, the soil displacement due to sheet and rill erosion in US croplands was estimated to be 1.59 Pg yr^−1^ in 1982 and 0.96 Pg yr^−1^ in 2012. The GloSEM estimates for soil erosion in US croplands during the same periods (considering the annual conditions of land use and CA) were equal to 1.52 and 0.91 Pg yr^−1^ in 1982 and 2012, respectively. The deviation between the statistical data of the USDA and our model estimates was, in both cases, below 5%. The second regional comparison was carried out for the cropland of the European Union (EU) using the higher-spatial-resolution (25-m) RUSLE-based model, developed by the European Commission (EC)^44^. Here, the comparison also revealed a consistency in soil erosion estimates for the EU cropland in the selected reference year 2012 (GloSEM = 0.304 Pg yr^−1^; EC RUSLE2015 = 0.30 Pg yr^−1^). In this second case, we also noted a minor deviation (below 5%) between the GloSEM and regional-scale estimates. Concerning 2019, the combination of the GloSEM 1.3 soil displacement rates with the country cropland statistics (US = 0.36 billion ha [US census of agriculture 2017]; EU = 0.17 billion ha [Eurostat 2018]) yielded estimates of 0.99 Pg yr^−1^ for the US and 0.3 Pg yr^−1^ for the EU croplands. Both of these are in line with the regional estimates provided by the NRI for the US (0.98 Pg yr^−1^) (USDA^45^) and the EC for the EU28 (0.3 Pg yr^−1^)^44^. The good agreement between our estimates and the ones provided by independent studies give confidence that the quantitative estimates achieved through the global model are reliable and valid to a level close to the higher-resolution regional assessments.

### Evaluation of the GPR forecasting capacity

In order to test the GPR forecasting capacity, a model was fitted on the pre-2000 data using the estimated rainfall erosivity (R-)factor derived from measured data (dependent variable) and WorldClim historical data (averaged over the same timespan). The fitted GPR model was subsequently used to predict the values for the post-2000 period using the WorldClim data for the same time horizon. This procedure constitutes a form of external validation in which the validation dataset belongs to a different period. The procedure resulted in a very good prediction capacity for the pre-2000 training set (0.85 R^2^) and a good prediction capacity for the post-2000 validation set (0.6 R^2^). Note, however, that the model tended to be less effective with data with very high R-factor values, which mainly appeared at >10,000 MJ mm h^−1^ ha^−1^ yr^−1^. Here, the model tended to underestimate the predicted R-factor values. Of further note was that the resolution of the WorldClim data is rather coarse, and this might result in a mismatch between station-measured data and WorldClim data, especially in areas where the topography strongly influences precipitation. Moreover, the two datasets are spatially mismatched, meaning that the stations from the pre-2000 dataset do not necessarily overlap those of the post-2000 dataset. This introduced more error because the model lacked full spatial coverage, allowing it to extrapolate to more diverse climatic conditions. Nevertheless, the model showed little bias when predicting the post-2000 R-factor values, which were comparable to those of the training data (pre-2000).

## Usage Notes

The provided layers express rates of soil displacement by water erosion, expressed as a mass of soil lost per unit area and time (Mg ha^−1^ yr^−1^), given by the local combination of climate, soil, topography, farming and management system. These estimates do not account for the fractional (%) cropland cover of each cell. The total soil displacement of each scenario can be estimated by combining the provided soil erosion rate with the fractional (%) cropland cover layer (ESA100_Cropland_fraction.tif). This operation may require the reprojection of the (WGS84) geographic data to a local or global state-plane projection.

## Supplementary information

Table S1

## Data Availability

No custom code was used to generate or process the data.
