# Supplementary material for: GloSEM: High-resolution global estimates of present and future soil displacement in croplands by water erosion
Source: Sci Data. 2022 Jul 13;9:406. doi: 10.1038/s41597-022-01489-x (PMC9279367; doi:10.1038/s41597-022-01489-x)
Supplement: Supplementary file 1 — Table S1 [file 41597_2022_1489_MOESM1_ESM.docx]

### Supplementary Information

**Table S1. Country-based descriptive statistics of soil displacement estimates for the 2019 and 2070 scenarios.**

| **Country** | **Area (ha)** | **Cropland (%)** | **Displacement rate (Mg ha^-1^ yr^-1^)** | | | |
| --- | --- | --- | --- | --- | --- | --- |
|  |  |  | **2019** | **2.6** | **4.5** | **8.5** |
| Afghanistan | 4,545,770 | 35.8 | 3.2 | 5.7 | 6.3 | 7.2 |
| Aland Islands | 8,270 | 24.4 | 0.5 | 0.7 | 0.9 | 1.2 |
| Albania | 425,871 | 24.1 | 21.9 | 24.6 | 25.7 | 26.5 |
| Algeria | 6,003,861 | 35.9 | 2.8 | 2.7 | 3.0 | 3.0 |
| Andorra | 90 | 9.3 | 41.8 | 58.7 | 57.9 | 53.1 |
| Angola | 7,905,552 | 22.6 | 21.4 | 23.1 | 23.6 | 24.0 |
| Antigua and Barbuda | 3,045 | 18.1 | 25.4 | 20.5 | 15.9 | 14.7 |
| Argentina | 40,106,366 | 56.1 | 5.3 | 6.5 | 7.0 | 7.2 |
| Armenia | 500,723 | 43.7 | 2.5 | 3.8 | 3.7 | 4.4 |
| Australia | 31,308,271 | 43.0 | 1.5 | 1.6 | 1.7 | 1.9 |
| Austria | 1,516,213 | 40.1 | 7.4 | 10.7 | 11.6 | 13.3 |
| Azerbaijan | 2,839,744 | 51.0 | 2.7 | 2.8 | 2.8 | 3.1 |
| Bahamas | 39,725 | 17.4 | 7.6 | 6.7 | 5.9 | 5.5 |
| Bahrain | 1,266 | 18.4 | 0.7 | 1.0 | 1.2 | 1.3 |
| Bangladesh | 6,514,030 | 54.2 | 12.9 | 12.2 | 12.3 | 13.5 |
| Barbados | 6,108 | 23.2 | 15.7 | 13.8 | 12.4 | 11.5 |
| Belarus | 6,989,666 | 43.2 | 0.6 | 0.9 | 0.9 | 1.1 |
| Belgium | 1,112,843 | 43.1 | 1.7 | 2.7 | 2.9 | 3.6 |
| Belize | 207,170 | 64.3 | 22.1 | 20.9 | 19.8 | 18.4 |
| Benin | 3,028,152 | 34.0 | 12.4 | 18.6 | 19.2 | 19.6 |
| Bhutan | 61,855 | 13.7 | 101.4 | 99.0 | 100.8 | 105.9 |
| Bolivia | 2,035,284 | 25.5 | 18.8 | 21.1 | 21.8 | 21.6 |
| Bosnia & Herzegovina | 734,009 | 25.8 | 17.0 | 17.5 | 18.2 | 19.5 |
| Botswana | 1,800,346 | 21.6 | 4.3 | 4.3 | 3.9 | 3.9 |
| Brazil | 95,300,952 | 32.2 | 29.2 | 32.3 | 33.8 | 33.6 |
| British Virgin Islands | 547 | 13.0 | 60.9 | 54.1 | 46.3 | 43.1 |
| Brunei Darussalam | 2,030 | 9.1 | 27.7 | 47.5 | 48.6 | 48.4 |
| Bulgaria | 4,575,295 | 55.9 | 4.4 | 5.2 | 5.5 | 5.6 |
| Burkina Faso | 8,241,912 | 33.5 | 5.9 | 9.3 | 9.6 | 10.0 |
| Burundi | 748,724 | 30.7 | 62.2 | 59.1 | 66.9 | 72.1 |
| Cambodia | 5,651,820 | 46.9 | 11.7 | 14.1 | 14.2 | 13.7 |
| Cameroon | 2,780,054 | 29.0 | 23.4 | 29.4 | 30.3 | 31.1 |
| Canada | 34,653,410 | 48.4 | 1.2 | 2.0 | 2.3 | 2.5 |
| Cayman Islands | 170 | 11.6 | 11.6 | 12.3 | 11.0 | 10.4 |
| Central African Rep. | 337,147 | 20.1 | 15.9 | 24.3 | 25.0 | 26.0 |
| Chad | 9,838,226 | 21.9 | 3.4 | 5.6 | 5.8 | 5.9 |
| Chile | 2,816,583 | 42.6 | 6.3 | 7.6 | 7.0 | 6.5 |
| China | 132,318,658 | 38.4 | 22.2 | 27.0 | 28.7 | 31.2 |
| Colombia | 2,350,045 | 26.3 | 46.1 | 46.8 | 47.4 | 50.3 |
| Comoros | 9,520 | 17.6 | 177.4 | 175.8 | 172.5 | 169.8 |
| Costa Rica | 1,227,943 | 60.7 | 61.9 | 73.7 | 73.2 | 72.6 |
| Cote d'Ivoire | 1,997,922 | 15.1 | 15.7 | 21.4 | 21.5 | 22.0 |
| Croatia | 1,376,027 | 37.2 | 8.9 | 10.5 | 11.0 | 11.7 |
| Cuba | 906,237 | 22.3 | 15.0 | 16.7 | 14.5 | 13.8 |
| Cyprus | 293,483 | 49.9 | 2.5 | 2.8 | 2.5 | 1.7 |
| Czech Republic | 3,355,523 | 54.8 | 2.2 | 3.2 | 3.6 | 4.3 |
| North Korea | 2,329,134 | 34.7 | 59.4 | 74.0 | 76.0 | 80.5 |
| Democratic Rep. Congo | 4,003,068 | 21.1 | 50.4 | 52.4 | 56.0 | 58.4 |
| Denmark | 2,377,017 | 61.2 | 0.9 | 1.1 | 1.2 | 1.5 |
| Djibouti | 11,531 | 13.9 | 16.5 | 22.0 | 21.0 | 25.6 |
| Dominica | 576 | 9.3 | 169.3 | 174.6 | 172.6 | 171.5 |
| Dominican Republic | 269,894 | 18.0 | 27.5 | 31.4 | 28.3 | 27.3 |
| Ecuador | 1,542,677 | 27.7 | 39.3 | 27.3 | 29.0 | 33.1 |
| Egypt | 3,268,236 | 61.3 | 0.2 | 1.3 | 1.5 | 1.6 |
| El Salvador | 623,734 | 52.7 | 66.4 | 93.5 | 92.6 | 88.9 |
| Equatorial Guinea | 10,091 | 11.2 | 77.7 | 84.2 | 84.8 | 86.1 |
| Eritrea | 839,109 | 22.5 | 21.9 | 36.0 | 34.2 | 35.8 |
| Estonia | 1,092,498 | 34.9 | 0.7 | 0.7 | 0.8 | 1.0 |
| Ethiopia | 17,170,993 | 31.5 | 47.5 | 61.8 | 64.1 | 66.8 |
| Fiji | 61,695 | 14.3 | 109.5 | 121.2 | 117.2 | 113.6 |
| Finland | 874,430 | 26.1 | 0.4 | 0.4 | 0.5 | 0.7 |
| France | 22,381,781 | 49.0 | 3.2 | 4.8 | 5.4 | 5.9 |
| French Guiana | 1,498 | 11.7 | 36.0 | 41.4 | 40.6 | 39.1 |
| Gabon | 144,879 | 12.0 | 39.7 | 59.6 | 59.7 | 58.6 |
| Gambia | 269,663 | 31.3 | 5.3 | 7.3 | 6.9 | 6.6 |
| Georgia | 896,513 | 40.0 | 8.3 | 10.6 | 11.1 | 12.9 |
| Germany | 14,978,466 | 52.7 | 1.4 | 2.2 | 2.5 | 3.1 |
| Ghana | 3,322,615 | 24.6 | 14.0 | 19.5 | 20.0 | 20.8 |
| Gibraltar | 2 | 23.9 | 16.9 | 15.4 | 12.7 | 9.8 |
| Greece | 2,297,835 | 34.1 | 6.8 | 6.7 | 6.5 | 6.1 |
| Grenada | 422 | 11.9 | 125.0 | 126.2 | 121.9 | 117.4 |
| Guadeloupe | 5,396 | 16.2 | 19.8 | 19.0 | 16.4 | 15.7 |
| Guatemala | 2,582,070 | 57.2 | 59.9 | 66.3 | 64.1 | 64.4 |
| Guinea | 873,319 | 16.4 | 19.5 | 28.1 | 28.5 | 28.9 |
| Guinea-Bissau | 152,909 | 14.0 | 6.7 | 10.8 | 10.8 | 10.6 |
| Guyana | 42,899 | 17.0 | 7.6 | 10.6 | 10.0 | 9.8 |
| Haiti | 198,421 | 14.4 | 92.9 | 105.4 | 100.3 | 98.0 |
| Honduras | 1,760,632 | 56.0 | 72.2 | 81.6 | 77.8 | 78.3 |
| Hong Kong | 501 | 15.6 | 143.5 | 154.6 | 154.4 | 155.3 |
| Hungary | 5,395,949 | 63.7 | 1.8 | 2.5 | 2.7 | 3.1 |
| Iceland | 75,139 | 20.6 | 6.9 | 8.1 | 8.5 | 8.1 |
| India | 146,132,869 | 58.2 | 10.4 | 11.8 | 12.1 | 12.9 |
| Indonesia | 6,709,782 | 21.1 | 35.7 | 43.0 | 43.3 | 42.8 |
| Iran | 17,499,648 | 45.0 | 1.2 | 1.9 | 2.0 | 2.5 |
| Iraq | 10,670,287 | 51.4 | 1.6 | 3.1 | 3.1 | 3.4 |
| Ireland | 2,218,442 | 35.4 | 3.7 | 4.2 | 4.4 | 5.1 |
| Israel | 396,325 | 46.5 | 2.8 | 2.2 | 1.5 | 0.9 |
| Italy | 6,787,771 | 34.2 | 10.8 | 12.7 | 12.6 | 12.0 |
| Jamaica | 24,674 | 16.0 | 33.5 | 32.8 | 30.8 | 30.0 |
| Japan | 2,787,358 | 26.2 | 35.5 | 38.6 | 40.7 | 43.1 |
| Jordan | 369,275 | 36.8 | 1.4 | 1.8 | 1.4 | 1.0 |
| Kazakhstan | 31,362,313 | 36.2 | 0.6 | 0.7 | 0.9 | 1.0 |
| Kenya | 7,650,383 | 28.0 | 26.0 | 33.4 | 36.7 | 38.2 |
| Kuwait | 32,401 | 29.5 | 0.3 | 1.8 | 1.9 | 2.0 |
| Kyrgyzstan | 1,025,394 | 27.8 | 5.6 | 8.3 | 9.5 | 10.7 |
| Lao PDR | 1,618,426 | 31.8 | 27.8 | 27.7 | 29.6 | 29.5 |
| Latvia | 1,800,603 | 38.8 | 0.9 | 0.9 | 1.0 | 1.3 |
| Lebanon | 163,852 | 32.2 | 8.5 | 9.5 | 8.8 | 8.4 |
| Lesotho | 455,141 | 31.4 | 49.7 | 56.8 | 60.5 | 64.5 |
| Liberia | 22,225 | 7.5 | 27.4 | 36.8 | 36.9 | 36.5 |
| Libya | 1,859,114 | 37.8 | 0.5 | 1.2 | 1.1 | 0.9 |
| Liechtenstein | 1,333 | 27.2 | 6.9 | 10.6 | 11.6 | 13.5 |
| Lithuania | 2,602,067 | 48.4 | 0.7 | 0.7 | 0.8 | 1.0 |
| Luxembourg | 89,050 | 38.0 | 3.9 | 4.4 | 4.9 | 6.4 |
| Madagascar | 8,215,934 | 21.3 | 42.9 | 43.5 | 43.5 | 43.3 |
| Malawi | 2,606,133 | 29.7 | 35.2 | 32.2 | 33.2 | 33.0 |
| Malaysia | 620,643 | 20.7 | 20.9 | 27.8 | 28.1 | 27.5 |
| Maldives | 351 | 23.0 | 11.2 | 17.0 | 17.5 | 17.1 |
| Mali | 13,196,840 | 28.3 | 4.8 | 6.6 | 6.7 | 6.7 |
| Malta | 6,907 | 28.8 | 11.1 | 6.0 | 4.8 | 5.1 |
| Martinique | 1,581 | 12.4 | 74.1 | 85.2 | 79.7 | 77.4 |
| Mauritania | 2,114,005 | 20.6 | 2.4 | 2.9 | 2.8 | 3.0 |
| Mauritious | 18,630 | 45.8 | 83.3 | 89.0 | 86.7 | 84.0 |
| Mexico | 20,402,781 | 33.8 | 24.0 | 26.6 | 26.5 | 25.9 |
| Mongolia | 3,411,521 | 26.4 | 1.9 | 1.9 | 2.1 | 2.3 |
| Montenegro | 139,572 | 18.1 | 37.0 | 33.3 | 34.4 | 35.2 |
| Montserrat | 334 | 14.4 | 160.5 | 160.6 | 151.0 | 148.4 |
| Morocco | 6,082,550 | 40.9 | 4.9 | 6.1 | 5.2 | 5.0 |
| Mozambique | 9,101,146 | 19.0 | 20.4 | 19.8 | 20.5 | 19.9 |
| Myanmar | 10,565,370 | 42.5 | 17.2 | 20.8 | 22.1 | 22.7 |
| Namibia | 1,299,756 | 22.4 | 3.4 | 2.9 | 2.8 | 2.8 |
| Nepal | 2,202,691 | 33.9 | 62.4 | 67.7 | 68.1 | 70.8 |
| Netherlands | 1,375,932 | 47.3 | 0.5 | 0.9 | 1.0 | 1.2 |
| New Caledonia | 36,307 | 9.5 | 89.9 | 88.7 | 89.8 | 88.7 |
| New Zealand | 2,628,096 | 34.2 | 7.1 | 9.1 | 9.9 | 11.1 |
| Nicaragua | 3,227,562 | 62.4 | 69.7 | 79.8 | 78.0 | 78.4 |
| Niger | 8,279,540 | 29.4 | 2.2 | 3.2 | 3.2 | 3.4 |
| Nigeria | 25,198,943 | 34.4 | 11.6 | 16.7 | 17.1 | 17.8 |
| Norway | 507,472 | 17.6 | 21.8 | 30.6 | 32.1 | 34.6 |
| Occ. Palestinian Ter. | 109,657 | 28.0 | 4.3 | 3.5 | 2.5 | 1.3 |
| Oman | 105,697 | 20.0 | 2.8 | 5.7 | 5.9 | 6.2 |
| Pakistan | 17,816,898 | 55.9 | 5.8 | 7.5 | 7.6 | 7.4 |
| Panama | 1,208,559 | 57.1 | 52.1 | 68.2 | 68.7 | 67.7 |
| Papua New Guinea | 197,828 | 7.8 | 79.7 | 92.4 | 93.4 | 92.8 |
| Paraguay | 5,887,491 | 37.4 | 14.4 | 16.6 | 17.9 | 17.6 |
| Peru | 2,530,347 | 19.7 | 46.2 | 58.4 | 60.5 | 64.5 |
| Philippines | 3,454,499 | 29.8 | 45.3 | 52.9 | 53.9 | 54.0 |
| Poland | 13,305,186 | 53.8 | 1.0 | 1.4 | 1.5 | 1.9 |
| Portugal | 1,477,556 | 28.8 | 3.8 | 4.2 | 4.2 | 4.3 |
| Puerto Rico | 19,880 | 14.7 | 35.8 | 39.2 | 34.4 | 32.3 |
| Qatar | 17,105 | 26.6 | 0.4 | 0.7 | 0.8 | 1.0 |
| Republic of Korea | 1,446,292 | 31.8 | 52.0 | 59.4 | 60.3 | 62.4 |
| Republic of Moldova | 2,066,839 | 63.3 | 3.6 | 5.8 | 6.0 | 6.5 |
| Republic of the Congo | 524,091 | 14.5 | 41.6 | 55.6 | 55.5 | 54.9 |
| Romania | 10,056,509 | 54.5 | 3.5 | 5.2 | 5.2 | 5.8 |
| Russian Federation | 100,420,173 | 44.15 | 1.3 | 1.9 | 2.1 | 2.5 |
| Rwanda | 726,844 | 32.1 | 72.1 | 71.2 | 78.0 | 84.4 |
| Saint Barthelemy | 1 | 13.8 | 52.6 | 38.1 | 31.7 | 30.8 |
| Saint Kitts and Nevis | 1,393 | 14.6 | 46.7 | 41.7 | 33.6 | 32.2 |
| Saint Lucia | 579 | 13.0 | 74.5 | 76.3 | 71.7 | 68.8 |
| Saint Martin | 239 | 14.0 | 71.7 | 55.0 | 45.4 | 43.1 |
| Saint Vincent | 753 | 12.8 | 138.3 | 142.5 | 137.1 | 132.7 |
| San Marino | 1,277 | 25.0 | 25.3 | 28.1 | 29.9 | 28.2 |
| Sao Tome and Principe | 1,041 | 14.4 | 84.8 | 122.3 | 122.0 | 123.5 |
| Saudi Arabia | 1,992,633 | 33.1 | 0.7 | 4.5 | 4.7 | 4.8 |
| Senegal | 4,561,835 | 31.8 | 3.4 | 4.6 | 4.4 | 4.2 |
| Serbia | 3,110,419 | 50.0 | 4.5 | 5.2 | 5.6 | 6.1 |
| Seychelles | 1,338 | 18.2 | 7.3 | 8.2 | 7.8 | 7.8 |
| Sierra Leone | 147,711 | 14.2 | 19.0 | 27.4 | 28.2 | 29.3 |
| Singapore | 1,957 | 14.7 | 31.7 | 39.3 | 39.0 | 37.7 |
| Slovakia | 1,911,766 | 46.7 | 4.7 | 7.2 | 7.9 | 8.8 |
| Slovenia | 284,787 | 27.2 | 21.4 | 28.5 | 28.2 | 29.6 |
| Solomon Islands | 1,384 | 7.1 | 70.1 | 84.8 | 84.5 | 81.2 |
| Somalia | 1,637,775 | 24.8 | 3.3 | 7.2 | 7.2 | 7.6 |
| South Africa | 16,486,529 | 34.0 | 15.3 | 16.1 | 16.2 | 16.7 |
| Spain | 12,585,246 | 39.6 | 3.7 | 4.3 | 4.2 | 3.9 |
| Sri Lanka | 920,127 | 25.7 | 12.4 | 19.1 | 19.4 | 19.3 |
| Sudan | 16,080,094 | 26.5 | 2.4 | 4.2 | 4.3 | 4.3 |
| Suriname | 10,446 | 14.5 | 10.3 | 10.5 | 10.1 | 10.0 |
| Swaziland | 447,030 | 37.4 | 32.7 | 31.1 | 30.6 | 32.5 |
| Sweden | 1,869,562 | 29.9 | 0.9 | 1.1 | 1.2 | 1.6 |
| Switzerland | 498,677 | 30.0 | 14.2 | 19.2 | 21.0 | 23.2 |
| Syrian Arab Republic | 6,776,858 | 55.0 | 1.0 | 1.2 | 1.2 | 1.2 |
| Taiwan | 344,538 | 29.7 | 40.1 | 48.7 | 48.8 | 49.4 |
| Tajikistan | 1,222,587 | 36.3 | 13.4 | 17.7 | 21.4 | 25.8 |
| Thailand | 14,204,328 | 48.8 | 13.2 | 15.5 | 16.2 | 15.5 |
| Macedonia | 496,128 | 31.6 | 7.6 | 8.0 | 8.7 | 8.5 |
| Timor-Leste | 84,390 | 10.7 | 114.4 | 116.1 | 115.6 | 116.1 |
| Togo | 1,383,315 | 34.2 | 15.6 | 22.4 | 22.7 | 23.4 |
| Tonga | 2,956 | 23.9 | 38.2 | 36.1 | 36.7 | 35.5 |
| Trinidad and Tobago | 11,214 | 16.1 | 20.6 | 21.2 | 19.3 | 18.3 |
| Tunisia | 2,033,819 | 32.7 | 2.3 | 2.7 | 2.6 | 2.3 |
| Turkey | 19,828,964 | 50.1 | 2.4 | 2.7 | 3.0 | 3.2 |
| Turkmenistan | 5,317,591 | 43.8 | 1.8 | 2.2 | 2.4 | 2.8 |
| Uganda | 4,339,840 | 28.1 | 27.0 | 27.7 | 31.4 | 33.7 |
| Ukraine | 32,462,938 | 62.2 | 1.3 | 2.1 | 2.2 | 2.5 |
| United Arab Emirates | 50,691 | 19.0 | 0.6 | 0.9 | 1.0 | 1.2 |
| United Kingdom | 9,998,969 | 46.9 | 6.7 | 9.4 | 10.1 | 11.2 |
| Tanzania | 23,190,093 | 30.8 | 22.7 | 20.7 | 22.5 | 22.5 |
| United States | 137,822,995 | 43.6 | 7.2 | 10.6 | 11.4 | 12.1 |
| Uruguay | 3,296,345 | 27.0 | 7.6 | 11.0 | 11.7 | 12.5 |
| Uzbekistan | 5,066,626 | 42.6 | 2.1 | 2.7 | 3.4 | 4.1 |
| Vanuatu | 6,772 | 8.3 | 102.2 | 127.0 | 125.7 | 121.5 |
| Venezuela | 2,032,723 | 26.2 | 12.1 | 15.9 | 14.5 | 14.5 |
| Viet Nam | 5,167,344 | 35.8 | 32.5 | 35.1 | 35.6 | 35.5 |
| Western Sahara | 1,052 | 6.3 | 0.3 | 1.1 | 1.2 | 1.3 |
| Yemen | 1,242,053 | 24.8 | 8.8 | 17.2 | 17.1 | 18.9 |
| Zambia | 8,486,974 | 21.1 | 17.4 | 17.2 | 17.5 | 17.0 |
| Zimbabwe | 9,307,277 | 28.6 | 20.5 | 19.4 | 19.1 | 18.0 |
